# AN INTERDISCIPLINARY APPROACH TO ADDRESSING HEARING LOSS IN THE INPATIENT SETTING TO IMPROVE COMMUNICATION

**DOI:** 10.1093/geroni/igad104.1470

**Published:** 2023-12-21

**Authors:** Nicholas Reed

**Affiliations:** Johns Hopkins Bloomberg School of Public Health, Baltimore, Maryland, United States

## Abstract

Nearly half of all adults over 60 years of age have hearing loss which is associated with decreased satisfaction with care, higher risk of readmission, and longer length of stay. These associations may be mediated by the barriers hearing loss places on patient-provider communication. There is a paucity of sustainable programs focused on addressing hearing loss among older adults in the inpatient. An interdisciplinary collaborative team comprising audiologists, geriatricians, and nurses developed and implemented a quality initiative to address hearing loss in the inpatient setting in two medical inpatient units at Johns Hopkins Bayview Medical Center that started with offering 16 training session slots to raise awareness of hearing loss and teach technologic, environmental, and communication considerations for adults with hearing loss. Over a 3-month period, 543 of 644 admitted patients were screened for hearing loss using a sustainable model built into the admission process. Those without hearing loss (52.6%) received no intervention, those with mild loss (31.3%) received signage in the room reminding providers of training, and those with moderate or greater loss (16.1%) received signage and an amplifier. At discharge, 79% and 81.6% of those with mild and moderate hearing loss indicated improved communication during their current stay relative to previous stays. Notably, a larger proportion of those without hearing loss (52.9%) also indicated improved communication. A post study survey among 18 nurses suggested themes of moderate (33%) desiring more training and overwhelming perceptions (94.4%) the program didn’t interfere with other duties and made communication with patients easier.

